# Caesarean section on maternal request - a qualitative study of stakeholders´ views

**DOI:** 10.1186/s12978-025-02206-8

**Published:** 2025-11-28

**Authors:** Maria Johansson Offerman, Inger K. Holmström, Mio Fredriksson, Heléne Appelgren Engström, Magdalena Mattebo

**Affiliations:** 1 School of Health, Care and Social Welfare, Division of Caring Sciences and Health Care Pedagogics, Mälardalen University, Box 883, Västerås, 721 23 Sweden; 2 Department of Public Health and Caring Sciences, Health Services Research, Uppsala University, Box 564, Uppsala, 751 22 Sweden

**Keywords:** Caesarean section on maternal request, Childbirth, Autonomy, Qualitative method, Reflexive thematic analysis

## Abstract

**Background:**

Caesarean section on maternal request (CSMR) raises ethical and clinical challenges despite Sweden’s low overall caesarean section (CS) rate. National written recommendations are restrictive, yet regional differences suggest unequal care. While previous research has focused on women and healthcare professionals, little is known about the views on CSMR of policymakers and other key stakeholders. The aim of this study was to investigate how stakeholders in the Swedish healthcare system view CSMR in relation to medical considerations, individual autonomy and societal values.

**Methods:**

A qualitative study with an inductive approach was conducted using reflexive thematic analysis of semi-structured interviews. Sixteen stakeholders were recruited, including regional politicians, policymakers, national authority representatives, and members from organisations and associations with relevant interest and expertise in the field.

**Results:**

Five themes were generated: (1) Caesarean section is a valid way of giving birth for some; (2) The right to choose and the decision process are complex issues; (3) Individual options for childbirth are desirable; (4) There is a lack of trust in maternity care; (5) Economic and ethical challenges in maternity care exist. The participants viewed CSMR as a legitimate option for some women, despite the increased medical risks, which were considered concerning but not disqualifying. Support and guidance in decision-making were considered essential by the participants. They valued continuity in care and emotional support highly. The participants expressed the views that distrust in Swedish maternity care was linked to media portrayals and inconsistent handling of CSMR. Economic and ethical concerns included questions of resource allocation and the scope of public healthcare responsibilities. The option to pay privately for a planned CS was broadly rejected by the participants.

**Conclusions:**

This study highlights the complexity of CSMR and its varied perspectives. While individual risks may be low, population-level risks could rise with increased prevalence, and the perception of risk varies depending on perspective. Both medical and psychological risks should inform decisions, with counselling seen as crucial by the participants. Continuity of midwife care models may offer an alternative to CSMR for some. Stakeholders are key to ensuring clear guidelines, equal care, and trust in the system.

## Abstract (plain language summary)

This study looks at how stakeholders in Sweden view caesarean sections that are requested by women without a medical reason, known as caesarean section on maternal request (CSMR). Although Sweden has a low overall rate of caesarean sections (CS), national rules are strict in comparison with many other countries, and care can vary depending on where in Sweden a woman lives. While earlier research on CSMR has mainly focused on women and healthcare professionals, this study explores the views of other stakeholders, such as politicians, policymakers, and others involved in shaping maternity care. Sixteen stakeholders were interviewed. The participants stated that while CSMR carries some medical risks, these risks alone should not prevent women from having the option of CSMR. They stressed the importance of support and counselling during the decision-making process. The participants also stated that consistent care from the same midwife during pregnancy and birth could help reduce fear and build trust, possibly reducing the number of CSMR. Paying privately for a CSMR was generally not supported. The study shows that CSMR is a complex issue. It highlights the need for clear guidelines, equal access to care, and a maternity system that supports both individual choice and population health.

## Background

Caesarean section (CS) as a way of giving birth has increased globally in the last few decades [1]. In 2018, the global CS rate was 21.1%, with the lowest rates in sub-Saharan Africa and the highest rates in Latin America and the Caribbean. In Europe, Sweden has one of the lowest CS rates, with an overall rate of 19.5% in 2023 [2]. The global increase in CS rates can be explained by different factors, such as cultural preferences among women, convenience for and attitudes among healthcare professionals, the organisation of the healthcare system, and the country’s financial status [3–6]. Other contributing factors may include a lower tolerance for any associated complications and an association between increased maternal age and CS [3, 7].

Reasons for a planned CS can be medical, such as placenta praevia, a breech presentation or cephalopelvic disproportion [8]. In most contexts, the medical risks associated with a planned CS are slightly higher for the woman compared to those of a vaginal birth. Examples of such risks include infection, excessive bleeding, pulmonary thrombosis after birth, and a higher risk of the placenta growing into the uterine wall in a subsequent pregnancy [8, 9]. However, there are also medical complications that can follow a vaginal birth that are eliminated or reduced with a planned CS, such as anal sphincter injury and, in the long term, urinary stress incontinence [8]. For the baby, a planned CS is associated with an increased risk of admission to the neonatal intensive care unit (NICU), an increased risk of respiratory morbidity after birth, and a higher likelihood of developing asthma after a planned CS compared to a vaginal birth [8]. Also, a planned CS is costlier compared to a vaginal birth, both in the short and long - terms [8].

A caesarean section on maternal request (CSMR) can be defined as a CS performed at the woman’s request, where no obstetrical or medical contraindications have been identified for a vaginal birth [10]. Reasons for a CSMR can include fear of labour pain, fear of childbirth, anxiety about foetal injury and death, fear of vaginal and pelvic floor trauma or urinary incontinence, experience of a prior traumatic birth, as well as factors such as anxiety about lack of support from professionals and loss of control [11]. Other factors that can be added include considering a planned CS as less stressful and safer compared to vaginal delivery, and having a positive attitude towards CSMR if peers also opt for it [12].

The global incidence of CSMR varies between 0.2 and 42% across countries [6]. In Sweden, the number of women who request a CS is uncertain, but approximately 1–2% of all primiparous births and 3–7% of all multiparous births are CSMR [8]. Healthcare professionals have described encounters with women requesting a planned CS as demanding and time-consuming, noting the challenge of balancing the needs of these women, the expectations of other healthcare professionals, and their responsibility towards society [8]. From the women’s perspective, the decision-making process has been described as arbitrary and frivolous, and a need to defend the request for a planned CS against the healthcare professionals has been described [8]. The current Swedish written recommendations on CSMR are restrictive [13]. Even though the number of women requesting or wanting a planned CS are uncertain, healthcare professionals’ descriptions highlight potential conflicts with delivering person-centred care, including the areas of shared decision-making, patient safety, and a restrictive approach to CSMR [14].

A woman has no right to demand a CS by law in Sweden, but healthcare should, as far as possible, be provided in agreement with the patient [15]. National medical indications on CSMR published in 2011 were developed to provide decision support for healthcare professionals and specify under what conditions a woman’s request for a CS can be accommodated [13]. To get a CSMR, the woman should report her reasons, and the reasons should be assessed as sufficiently compelling. The woman should adhere to her request after receiving information about the risks and consequences, and she should have been offered counselling. The national medical indications from 2011 also state that a restrictive approach should be adopted and that the maternity clinics should have a policy established for how women with different reasons and risk levels in regard to CSMR should be managed, with the aim of achieving standardisation among clinics [13]. Although the 2011 medical indications on CSMR was intended to standardise clinical practice through a restrictive framework, its implementation has been inconsistent. The document is a recommendation and non-binding subject to interpretation, resulting in varied local policies and unclear impact on clinical routines [16]. Sweden consists of 21 self-governing regions which are responsible for the funding and delivery of healthcare. This is one explanation for the substantial regional difference in both planned and emergency CS rates in Sweden. This may to some extent be caused by differences in local guidelines, both in terms of their content and in whether such guidelines exist at all, as some clinics have them and others do not [2, 16]. Thus, the current situation indicates that women who request CS are not offered just and equal care, although the Health and Medical Service Act stipulates care on equal terms for the entire population [15, 16].

Previous studies have illustrated different opinions among women and healthcare professionals about whether women should have the right to choose a CS. However, there is also a variation within the group of healthcare professionals in attitudes towards women’s right to choose a CS [8, 17–19]. What is considered a medical reason for CS also differs, for example, whether fear of birth is a medical reason or not. Furthermore, some healthcare professionals believe that women have the right to choose a CS if well informed while others believe that women do not have such a right [19]. Maternity care professionals’ own personal preferences have also been shown to influence the recommendations on mode of birth [18]. Women who request a CS on the other hand, might believe they have the right to choose their mode of birth and have their request for a CS accepted [18, 19].

Different risk evaluations between women and healthcare professionals have also been shown [8]. Studies show that women consider CS as the safest mode of birth, with little or no risk, while vaginal birth is regarded as riskier [19, 20]. In contrast, healthcare professionals believe that there are higher medical risks associated with CS compared to vaginal birth [19]. Attitudes towards CSMR among healthcare professionals differ in Sweden as well as in Europe and could be linked to cultural factors, the organisation of the perinatal care and legal liability [5].

Media portrayals of childbirth can influence women’s perceptions, contribute to the continued medicalisation of birth, and often lack representations of normal, uncomplicated births [21]. The debate about maternity care in Swedish media tends to focus on a constant state of crisis, highlighting a lack of midwives, a shortage of space for women giving birth, and inadequate care for childbirth injuries [22–24]. CSMR is portrayed in Swedish media as ethically complex, involving tensions around how risks are interpreted, who should make decisions, and whether individual preferences can and should be accommodated within a needs-based healthcare system [25].

Previous research has mainly focused on investigating the views on CSMR of women and healthcare professionals, including midwives and obstetricians. There is little research about the views of policymakers and other key stakeholders in the Swedish healthcare system, such as stakeholders in the regions responsible for the funding and provision of healthcare (politicians), national authorities, and organisations and associations with an interest and expertise in the field. Despite Sweden having one of the lowest CS rates in Europe, there is a significant knowledge gap and a lack of national consensus on how to handle CSMR. This knowledge gap risks leading to unequal care as well as reduced trust among both women and healthcare professionals. To address this knowledge gap, it is necessary to investigate and gather opinions from various stakeholders, including policymakers, politicians, national authorities, and organisations with expertise in the field. By understanding these stakeholders’ perspectives, we can develop or revise guidelines for CSMR and create national consensus, ensuring that decisions about CSMR are based on objective criteria rather than personal opinions and trends, and that care is provided on equal terms.

The aim of this study was to investigate how stakeholders in the Swedish healthcare system view CSMR in relation to medical considerations, individual autonomy and societal values.

## Methods

### Design

A qualitative descriptive study using an inductive approach was conducted.

### Sample and setting

The research team, including the experts within the field, collaboratively identified relevant organisations and individuals. This approach ensured that participants had professional relevance to the study topic and allowed for voluntary, informed participation without the involvement of gatekeepers. Participants were recruited via email using purposeful and snowball sampling techniques. Contact was made via publicly available email addresses, including both general accounts for organisations and associations, as well as some addresses belonging to individual representatives. When a general account for an organisation or association was used, interested individuals took the initiative to contact the first author directly via email to express their willingness to participate. The sample included stakeholders such as politicians in the regional assemblies, policymakers and representatives from national authorities, as well as individuals from organisations and associations with relevant interest and expertise in the field. They received information about the study, including its purpose, procedures, and data handling in a written information letter attached to the first e-mail. Information was given that participation was voluntary. If a stakeholder did not respond to the initial email, a follow-up email was sent as a reminder.

Overall, 16 participants were recruited. Of the participants, 13 were women and three were men. Four of the participants were politicians in the regions, responsible for the funding and provision of healthcare. Eight of the participants had a healthcare professional background, and some worked partly in maternity care, but all were recruited as participants due to their roles as stakeholders, as described above. Another four participants with different functions were also recruited.

### Data collection

The data collection took place between May and September 2024. The location and time for each interview were decided in dialogue with the participants and were based on their preferences. One interview was conducted face-to-face, two by telephone and 13 online via Teams.

The interviews were semi-structured, conducted by the first author and lasted between 31 and 51 min. The interviews were recorded with a dictaphone and transcribed verbatim by the first author. The same interview guide was used for all interviews. The first interview was conducted as a pilot interview; no changes were made in the interview guide afterwards, and therefore the first interview was included in the study.

The concept of information power suggests that the adequacy of a qualitative sample is determined by the relevance and richness of the data, rather than the number of participants. Factors influencing information power include the aim of the study, specificity of the sample, quality of the interview dialogue, and the chosen method of analysis [26]. Sufficient information power, considering these factors, was deemed to have been achieved after 14 interviews. However, to ensure that all different perspectives were captured, two more interviews were conducted, resulting in 16 interviews overall. To assess the information power, the interviews were transcribed and reviewed during the process. Furthermore, we aimed for trustworthiness [27] through continuous documentation of the research process to ensure dependability, and a data management plan was created before the study started.

### Data analysis

The data was analysed using reflexive thematic analysis as described by Braun and Clarke [28] to identify patterns of shared meaning across the dataset. The method allows for a flexible approach, enabling the exploration of stakeholders’ multiple and diverse views on CSMR. The data analysis was inductive, focusing on the semantic content of the participants’ expressed views [28].

**Fig. 1:**
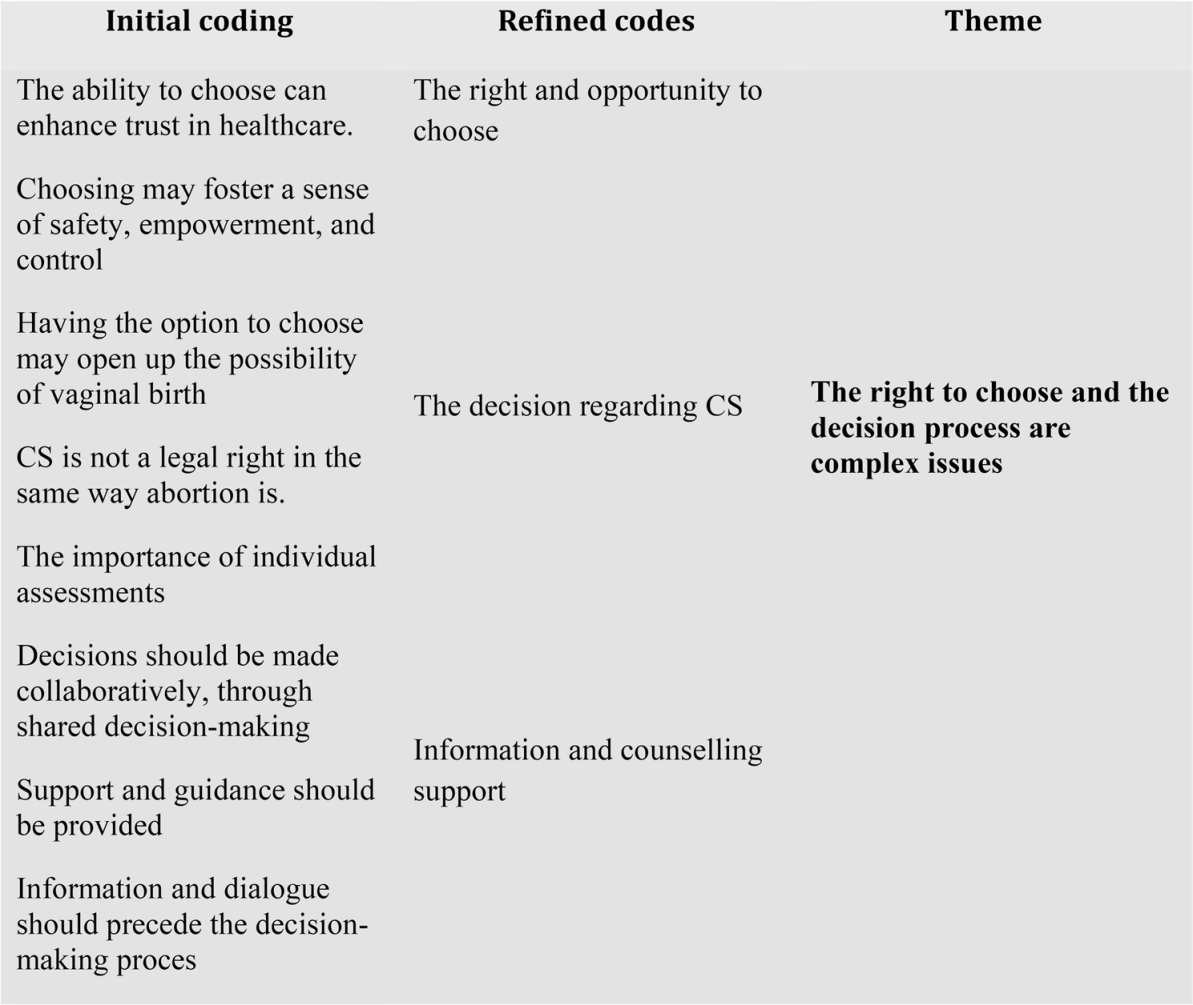
A visual representation of the reflexive thematic analysis process, as conceptualized by Braun and Clarke [28], illustrating the systematic organisation and synthesis of codes into a coherent theme

Following the six phases of reflexive thematic analysis described by Barun and Clarke [28], familiarisation with the dataset began with the transcription of the recorded interviews, and the transcripts were read multiple times. Initial manual coding was conducted by the first author and subsequently discussed with authors IKH, MF, HAE and MM. After two more rounds of refined coding, the first author and MM together reviewed and revised the codes from two different transcripts. An initial thematic map was then developed by the first author and discussed with all authors. Initial themes were generated, and in the next step, the thematic map and the themes were reviewed, refined and named in dialogue between all authors. The themes were repeatedly checked against the coded data and the transcribed dataset during the analysis (for example se Fig. 1). All interviews, transcriptions, coding and initial themes were in Swedish. Initially, the themes were refined, defined, and named in Swedish and subsequently translated into English by the authors. To secure rigour and enhance trustworthiness throughout the process several measures were taken. To achieve credibility, the research group´s different perspectives were reflected upon throughout the whole process. The analysis and interpretations were discussed within the research group as well as with other experts within the field involved in the project to ensure confirmability, and quotations were presented to support the analysis.

To further secure conformability we used triangulation when analysing data. The analysis was guided by a contextualist approach, as described by Braun and Clarke, acknowledging that while a reality exists, its meaning is shaped by context and interpretation [28]. Although the analysis was inductive and semantic, the researchers` positionalities influenced the interpretation, in line with reflexive thematic analysis. The first author, a licensed midwife and PhD student, has over a decade of experience in maternity care, including encounters with women requesting CSMR. This, along with the authors’ backgrounds in midwifery, nursing, and health policy, provides relevant pre-understanding shaped by clinical practice and the Swedish maternity care context.

### Ethical considerations

Ethical approval was obtained from the Swedish Ethical Review Authority (DNR: 2024-00637−01). The participants were informed about the study and its purpose before the interviews. Each interview began with recording verbal consent from the participants, and all participants also provided written consent. They were informed that participation was voluntarily, that they could withdraw without explanation at any time, and that all information would be treated confidentially and reported in such a way that the participants could not be identified. All data was stored securely on encrypted servers provided by Mälardalen University with access restricted to the research team. Data will be retained for 10 years in accordance with institutional and GDPR guidelines. Prior to analysis, all personal identifiers were removed, and data was anonymised. The process followed the ethical protocol approved by the Swedish Ethical Review Authority and participants were informed about how their data would be used, stored, and protected.

## Results

The views of the participants on CSMR in relation to medical considerations, individual autonomy and societal values generated five themes: (1) Caesarean section is a valid way of giving birth for some; (2) The right to choose and the decision process are complex issues; (3) Individual options for childbirth are desirable; (4) There is a lack of trust in maternity care; (5) Economic and ethical challenges in maternity care exist.

### Caesarean section is a valid way of giving birth for some

The participants believed that CSMR is a complex and difficult issue that could be examined from different perspectives; however, for some women, it should be a possible way of giving birth. Some participants stated that they believed women request CS because they see it as a safer way of giving birth. Another view that participants expressed was that vaginal birth is sometimes avoided by women as it is perceived to be less controlled, more unpredictable, and riskier for both the woman and child. Fears of pain, vaginal tears, and complications for the baby, as well as previous life experiences, experiences or trauma from a previous birth, mental health issues or psychiatric disorder were reasons that the participants believed were behind the woman’s request. For some women, a CSMR could be the best way of giving birth:

*If reduced stress is associated with a caesarean section*,* for example*,* if a woman has experienced vaginal birth trauma*,* it can be significantly less stressful to give birth via caesarean section. If the woman has experienced sexual abuse*,* it can be much easier to give birth via caesarean section*,* even though vaginal birth can sometimes be a fantastic form of redemption. (P5)*

Thoughts about self-determination regarding one’s own body were also brought up by the participants as a factor in the request for a CS. Some stated that psychological indications should carry the same weight as medical indications for CSMR. Some of the participants also stated that the possibility of a CS might be a prerequisite for some women to even dare to become pregnant and have children.

Most participants mentioned that the medical risks are greater with a CS compared to a vaginal birth, but some also stated that the medical risks with one or few CS are small for the individual woman and that the benefits of a CS can outweigh the disadvantages and risks. Most participants stated that the medical risks for the child are greater with a planned CS compared to vaginal birth, especially due to the increased risk of respiratory disorders. However, they did not consider this risk alone as a sufficient reason for denying CSMR. The view on risk differed among the participants, but they also stated that it could differ between health professionals and women. They believed that women tend to assess the risks of a vaginal birth as greater and the risks of a planned CS as smaller, compared to how the profession assesses them.

Some participants pointed out that there may be advantages to vaginal birth that the woman and child miss out on with a planned CS. These could be biological events and processes that are not fully understood at present but may be significant for individuals as well as for population health. Some participants described childbirth as one of life’s greatest events, with effects beyond the individual woman, and one participant expressed this as follows:

*I believe that childbirth*,* pregnancy*,* delivery*,* postpartum care*,* and parenting truly affect one’s entire life. They really do. A birth experience deeply impacts you and is part of you forever*,* and it can also set the tone for your parenting. This is not usually the case with a knee operation or a tooth extraction*,* which are often more isolated events. In this regard*,* I believe that those giving birth must be allowed to decide for themselves how they want it to be. (P4)*

Childbirth could hence be seen as having great impact and significance for the woman, her child and family and could, in the longer term, have a broader impact on society and population health.

### The right to choose and the decision process are complex issues

The right to choose and who makes the final decision regarding CSMR were also seen as complex issues. However, listening to the woman’s story and offering counselling and support were considered important. Most participants believed that it is not a woman’s right to choose a CS, but that being able to choose can strengthen trust in the healthcare services and in one’s own ability to make informed decisions. It can create a feeling of security, reduce stress, and give a sense of control. Having options and control in the situation can also, in some cases, open up the possibility of vaginal birth:

*In my experience*,* it is easier to encourage women to reconsider or contemplate a vaginal birth – and ultimately choose it – when they do not feel pressured*,* but rather when the decision is in their own hands. Unlike my colleagues who say no to a caesarean section and feel that the patient will change their mind and be happy with that decision*,* I think it´s actually that the patient wants to please and has given in. It is my own interpretation that when a woman gets a yes to a caesarean section*,* then she can sometimes reconsider. Because the woman is not cornered. No one else decides for you; now you decide for yourself. (P10)*

Participants held varying opinions on who should make the final decision regarding a CSMR. Some believed it should be the physician’s decision, others favoured a team of healthcare professionals, the women themselves, or ideally a shared decision-making process. However, most participants stated that a CSMR should not be approved lightly; it should not be a completely free choice but should be preceded by information and an individual assessment. The importance of individual assessments and that healthcare professionals should be open to the woman’s story and listen to her reasons was emphasised. Conversations should be held and information shared before the decision is made. Help and support should be provided as an opportunity for reflection and empowerment – not only before the birth but also in other aspects of life and in the transition to parenthood. As one participant stated:

*There are many women who do not seek treatment for their fear of childbirth but rather want a solution to what they perceive as the problem*,* which is to have a caesarean section. And I think it’s great that we can offer them treatment and support. There are many occasions in healthcare where you don’t get that opportunity. (P6)*

Some participants also articulated that it can be difficult to convey information, to ensure that the woman has received and understood the information, and that it can be difficult for women to evaluate the information about what a CS entails, especially from a long-term perspective.

### Individual options for childbirth are desirable

Maternity care that offers continuity and continuous support to the pregnant and labouring woman was seen as desirable by many of the participants, but not without obstacles. Whether it was realistic or possible in reality remained in doubt. It was pointed out by the participants that there are too few options in childbirth in today’s Swedish maternity care. Continuity of midwife care models and continuous support, including one-to-one midwifery support, during childbirth were consistently viewed positively by the participants. These were considered to create a sense of security, increase trust, and make some women want and dare to give birth vaginally. Continuity of midwife care models and continuous one-to one support were regarded as positive alternatives, especially in cases of fear of birth, mental health issues and psychiatric disorders. It was expressed as follows by one participant:

*I believe it can reduce the demand for planned caesarean sections. If you feel a sense of security related to the professional support you receive*,* you know the professionals*,* you have a good relationship*,* and you feel secure. And they can also help you feel competent. Women are usually able to give birth*,* and it usually goes very well. If you can get that feeling of confidence*,* then I think fewer would choose planned caesarean sections. (P7)*

However, the value of continuous support was considered to depend on it being provided by the right person, not necessarily a midwife. Higher costs, implementation challenges due to shortages of healthcare professionals, and a poorer working environment were cited by some participants as obstacles to care models that provide continuity of midwifery care and continuous one-to-one support.

Whether planned home birth services should be an option available in the public healthcare system was viewed from different perspectives. Some participants viewed planned home birth as an option that should be available for selected groups. Other participants believed that this should not be offered in public healthcare as it was not seen as medically safe, especially for the child, and posed risks in the case of an emergency. Higher costs and resource consumption for home births were also seen as obstacles.

Private options in maternity care, such as self-financed home births and private free-standing midwifery units where the woman pays for delivery care, were not considered something that should be banned. Many participants viewed the possibility of choice as fundamentally positive, stating that the intention behind such private options is good, and they can be seen as pioneers driving the development of care forward:

*And it can sometimes put a bit of pressure on the publicly funded healthcare system to actually develop when they see that women are seeking another alternative. This can lead to improvement and development since we don’t have differentiated care in Sweden today. There are very few alternatives. (P1)*

However, the participants also stated that the emergence of private options can be seen as a failure of public maternity care and may lead to unequal care. Concerns about the lack of medical safety and doubts about whether the need for these options really existed were other views from participants.

### There is a lack of trust in maternity care

A lack of trust in Swedish maternity care was believed to stem from reports of a crisis in Swedish maternity care and images and stories in traditional media and social media, as well as from perceived arbitrary and unequal handling of CSMR. The participants believed that the image of a crisis in maternity care affects trust and can lead to a desire for a planned CS. The media’s portrayal can induce anxiety and fear, and the participants considered the media’s image to be one-sidedly negative. They felt that many people want to share negative experiences on social media, and today many individuals find themselves in information and filter bubbles. One participant described the picture of the crisis in maternity care and what it can lead to as follows:

*I believe it affects the feeling of insecurity. Women may think*,* on the one hand*,* that they might not receive the good care they should. On the other hand*,* if something goes wrong*,* they might not get the necessary care either. You constantly read about women who struggle to get care related to childbirth injuries and who are not taken seriously*,* as everything looks fine on the surface. So*,* I think that the trust in maternity care*,* and its general image might be harmed. (P7)*

At the same time, stories and reports about deficiencies can lead to awareness and improvements. Healthcare professionals’ stories in the media about the crisis in maternity care can be seen as a cry for help, and the question arose as to where this debate should take place, publicly or within the profession. Many participants highlighted the importance of positive stories and examples and expressed a desire for more of them.

The need for clear national guidelines and routines was emphasised by the participants. The lack of national consensus in handling CSMR was described as potentially leading to personal or local values within the clinic prevailing in decision-making. This was considered to contribute to the perception of maternity care as unequal and arbitrary, leading to a lack of trust. One participant stated:

*So ideally*,* I think that such prioritisation decisions should be made at a higher level than in individual cases. That is*,* we should find some criteria for when a woman’s request is sufficient. Yes*,* when there are sufficiently strong reasons to accommodate the woman’s request. So*,* it’s clear*,* so it doesn’t become very different or up to whoever is making the decision. (P13)*

Clear national guidelines could help increase trust in maternity care among women and lead to the perception that care for CSMR is less arbitrary and unequal, according to the participants.

### Economic and ethical challenges in maternity care exist

Maternity care and CSMR present both economic and ethical challenges, including the question of the crowding out effect, short- and long-term economic impact, as well as the extent of public healthcare responsibilities. According to the participants, having the option to pay for a planned CS was not conceivable. A vast majority stated that this was the wrong direction for Swedish maternity care. Economics should not dictate decisions in maternity care, and providing tax-funded necessary care, such as care connected to pregnancy and childbirth, was considered an obligation and essential. At the same time, many participants noted that healthcare resources are limited and that economic considerations are inevitable. Paying for a planned CS was considered to potentially lead to unequal care. While it is currently possible in some areas to pay for other desired healthcare, the participants generally viewed this as inequitable and potentially leading to a decline in public healthcare:

*I think it’s the wrong way to go. Because this is a type of care that society has taken upon itself to offer*,* maternity care. And so there’s no reason why you should have to buy another type of care. So we’re talking about being able to buy your way past the healthcare queue and so on*,* and we shouldn’t have that in public healthcare as I see it. So*,* I think that we in public healthcare should be able to offer it. (P3)*

It was also mentioned that private options are not always medically safe, and that public healthcare then has the responsibility to manage and resolve complications and injuries. Comparisons were made with other types of surgery, such as plastic surgery performed abroad, where patients sometimes returned with injuries and complications that had to be managed by the public healthcare system. This was an outcome that most participants wished to avoid in the case of CSMR.

Some participants also mentioned that the long-term societal costs as a whole are difficult to assess and overview, such as the impact of childbirth on mental health, bonding, and parenting. The complex issue of how to assess the cost and consequences related to CSMR was expressed as follows by one participant:

*The economic factor matters in the short term*,* but in the long term*,* I think*,* it’s much harder to calculate. What does postpartum depression cost an entire family? What does it cost if bonding with the child doesn’t develop well in the long run? I think it costs society much more money than perhaps just the thousands for a caesarean section. But no one has really been able to calculate that. (P10)*

If more women give birth via planned CS, this could impact other types of care, as CS is a type of surgery that requires significant resources. It was agreed that resources in healthcare are limited, but the views differed on whether the limited resources in healthcare were something that could be affected by an increase in CSMR. Some participants believed there was a risk of crowding out effects. Others did not see this risk at all, considering it small, or believed that maternity care should not be compared to other parts of healthcare but should be assessed based on its own economic and resource conditions.

## Discussion

This study investigated how stakeholders in Sweden view CSMR in relation to medical considerations, individual autonomy and societal values. While previous research has mainly explored the views of women and healthcare professionals, including obstetricians and midwives, the perspectives of other stakeholders in the field have not been as thoroughly explored.

The views presented in the present study, that a planned CS could be the best way of giving birth for some women and that CSMR should be an option, have previously been shown [5, 18]. Previous research indicates a country variation in healthcare professionals’ views on support for CSMR, as well as differences based on gender and experience. Male obstetricians and less experienced obstetricians were more likely to support CSMR, and Swedish caregivers have generally taken a more restrictive approach compared to those in other countries [4, 5, 18]. Other findings in the present study indicate that the medical risks associated with a planned CS can be viewed from various perspectives. While some participants perceived the medical risks as significantly higher compared to vaginal births, others did not consider the medical risks of a single planned CS for an individual woman to be substantial. Even if the medical risks seem low for each individual, the growing number of CSMRs means that complications are likely to increase at the population level, which could make future births more difficult [19]. The views on risk and its valuation can differ depending on whether the perspective is individual or population-based. While CSMR may be beneficial for some women, at the population level it is essential to consider the increased complications from both medical and health-economic perspectives. The differences in perspectives, along with previous research indicating variations in attitudes towards CSMR based on gender and experience, underscore the importance of establishing clear guidelines and conducting individual assessments, both of which are equally crucial.

The impact that the experience of childbirth can have on a woman’s life was emphasised by the participants in the present study. The view was also expressed that it may affect attachment, society and population health from a broader perspective, in contrast to many other healthcare procedures or interventions. Associations have been observed between childbirth experience, which women recall years later, and different short- and long-term consequences such as exclusive breastfeeding, mental health status and sexual satisfaction [29]. Medical risks, particularly short-term risks, are in some respects easier to evaluate compared to psychological risks and the wider impact of childbirth. The participants in the present study stated that evaluating and managing these aspects are complex. Both medical and psychological dimensions are important to recognise and take into consideration in the decision-making process of CSMR. This indicates that a holistic approach that does not solely focus on medical outcomes can contribute to ensuring the long-term well-being of the woman, her child, her family and society.

The participants in the present study did not view the option to choose a planned CS without any limitations as a good solution. However, for some women, having the option to choose after receiving information and counselling was seen positively, as it could lead to a sense of control, reduced stress, and increased security. This has also been seen in studies where women describe a planned CS as a safer and less stressful way of giving birth [12, 19, 20]. Non-pregnant women, without children, who have a fear of birth, reported that the possibility of a planned CS gave them a sense of control. For some, being able to choose a planned CS made them open to attempting a vaginal birth [30]. This opinion was expressed by the participants in the present study, who also noted that if the decision was in the hands of the woman, some could reconsider and be open to trying a vaginal birth. This indicates that person-centred care, where individual needs and resources are identified, could be one way of approaching women with a fear of birth [31].

A conflict between the woman’s autonomy and right to make the final decision about CSMR, and the professional autonomy required to follow regulations, assess each case, and ultimately decide on CSMR, has previously been identified in interviews with healthcare professionals [14]. This conflict was not as evident in the perspectives of stakeholders in the present study, who are not directly involved in decisions for the individual woman. However, the conflict was recognised by some who still work in maternity care and were involved in the decision-making process for CSMR. The participants in the present study emphasised the importance of listening to the woman’s story, offering support, and providing counselling as a part of the decision-making process for CSMR. They stated that CSMR should not be offered without precautions. Counselling during the decision-making process was considered important, as it could not just help women handle the birth but could also provide support in other aspects of life. Previous research shows that for women who request a planned CS, acceptance of the request, rather than information and counselling, is the most important factor in their contact with healthcare professionals [8, 19]. This was also acknowledged by some participants in the present study who stated that women view a planned CS as a solution and do not ask for treatment or counselling. This could be seen as suggesting a potential conflict, where the participants in the present study view counselling and support as crucial and mandatory in the decision-making process for CSMR. In contrast, women may perceive such support from the perspective of their right to make autonomous decisions about their own body and health, without any prerequisites. The relatively low rate of CS in Sweden may, in part, reflect a cautious or restrictive approach among healthcare stakeholders regarding CSMR. This approach could potentially influence the degree of autonomy women experience in childbirth decision-making, particularly when individual preferences are weighed against institutional norms and guidelines.

The findings indicated that many participants in the present study viewed continuity of midwife care models and one-to-one support as positive and viable options. The World Health Organization (WHO) defines models featuring continuity of midwife care as models where “a known and trusted midwife, or a small group of known midwives, provides care to a woman and her baby throughout the antenatal, intrapartum and postnatal periods” [32]. These models are also referred to as caseload midwifery, midwifery-led continuity or team/midwifery group practice [32]. The participants in the present study primarily viewed the positive effects experienced by women, such as increased trust and security, as the main benefits of continuity of midwife care models and one-to-one support. Women with a fear of childbirth and psychiatric disorders were considered to especially benefit from these models. Almost all births in Sweden take place in hospitals, where the standard care involves the same midwife providing care during pregnancy, while hospital-employed midwives provide care and assistance during birth. Although continuity of midwife care models exists in Swedish maternity care, they are rare and only offered to a very limited group. A Cochrane review showed that continuity of midwife care models for low-risk women probably reduced CS, instrumental births, and episiotomies, while women reported more positive experiences [33]. A Swedish observational study found that women in a continuity of midwife care model were less likely to have a CSMR compared to women to women receiving standard care [34]. These models may lead to improvements in maternal depression, anxiety, and worry [35]. Women with a fear of childbirth, as well as midwives, reported that caseload midwifery generated a trustful relationship, which reduced fear, increased confidence, and contributed to a positive birth experience [36]. It is important to recognise the potential medical advantages of continuity of midwife care models and one-to-one support, but a positive birth experience is also crucial, not least for vulnerable groups, such as women with psychiatric disorders or a fear of childbirth. A sense of control, feelings of security, and reduced stress are factors associated with CSMR among women. Similar positive outcomes have also been linked to continuity of midwife care models, in which a trusting relationship is crucial to achieving a sense of control, feelings of security and reduced stress [36, 37]. Continuity of midwife care models may serve as an alternative to CSMR for some women by increasing control and trust and reducing fear and the risk of a negative birth experience. These models could be particularly important for vulnerable groups. By reducing the risk of a negative first birth experience, the number of multiparous women with a fear of birth considering CSMR could be decreased. Multiparas, who constitute the largest group considering CSMR, may also benefit from continuity of midwife care models. For some multiparas with a secondary fear of childbirth, stemming from their first birth experience, a model that ensures a trustful relationship, provides a higher sense of control, and reduces stress may serve as an alternative to CSMR.

The findings of the present study indicate that the participants perceived the image of Swedish maternity care as damaged and believed that there was a lack of trust among women in maternity care. This was due to stories and reports in the media, as well as the absence of national consensus on handling CSMR. How women engage in childbirth is influenced by how childbirth is represented and presented in media [21]. Swedish media presents CSMR as ethically complex, with tensions related to autonomy, risk, and justice, which may contribute to uncertainty and reduced trust among women [25]. There is a clear need to implement the current national guideline document or rewrite the various guidance documents existing in maternity clinics [16]. An individual assessment is always necessary, and guidelines cannot replace this with a complex issue like CSMR. However, they can function as a complement, and a structured approach can lay the foundation for just and equal care [16]. The media’s representation of maternity care as well as varying local guideline documents, or the lack thereof, play a crucial role in women’s perceptions and decisions regarding childbirth, potentially leading to an increase in the demand for CSMR. Enhancing communication between healthcare professionals and women, addressing fears and concerns, and offering support are crucial steps in rebuilding trust and repairing the damaged image of Swedish maternity care. In addition, the involvement of other stakeholders, such as those represented by the participants in the present study, in implementing clear national guidelines and ensuring just and equal care is equally important. The stakeholders, as represented by the participants in this study, bear a substantial and far-reaching responsibility for the organisation and structure of maternity care, as well as a corresponding capacity to influence its development. Their ability to initiate change and shape the discourse surrounding maternity care varies, yet collectively they have a pivotal role in driving the system towards reform.

The participants in the present study expressed concerns that an increase in CSMR could negatively affect other parts of the healthcare system. However, the idea that women themselves should be able to pay the additional cost of CSMR was not seen as an option. Almost all the participants believed that this would lead to unequal care and clearly stated that Swedish maternity care should not go in that direction. This was not considered a viable solution to the higher cost a planned CS entails, compared to a vaginal birth. A planned CS incurs higher costs in both the short and long term, and according to a recent report, the costs are higher at one, 10, and 20 years, considering the perspectives of both the woman and the child [8]. The importance of delivering equal care was considered fundamental by the participants. In accordance with the Health and Medical Service Act, it was deemed crucial that treatment and care be offered to the entire population on equal terms, with respect for equal value [15]. The importance of maintaining equal care within the Swedish healthcare system could be compromised if women were to pay the extra cost for a CSMR. This could result in a system where only those who can afford the additional cost would have access to this option, potentially deepening disparities in healthcare access and outcomes. Balancing individual needs and preferences, as well as the right to autonomy, with the broader implications for public healthcare is one of the main challenges associated with CSMR.

### Methodological considerations

A limitation of this study is its relatively small scale and that only three men were recruited to participate. An explanation for this could be that the majority of stakeholders in the field are women. Efforts were made to increase male participation; however, four additional men who were invited to take part either declined or did not respond. The Swedish healthcare system operates within a specific context, and the CS rates are low from a global perspective. Consequently, it may be difficult to transfer the findings to other settings with different healthcare systems. However, the participants were recruited from all over the country and represented different political parties, authorities, organisations and associations with interest and expertise in the field.

Most interviews were conducted online for convenience and due to the choice of participants. This approach allowed interviews to be conducted with participants from a wide geographical spread, at a time and place that suited them. All participants were accustomed to participating in online meetings, and no major technical problems occurred during the interviews. The topic was not sensitive, personal or likely to evoke distressing emotions, and the quality of the interviews is not assessed to have been affected by being conducted online [38]. The authors did not have any prior connections to the participants.

## Conclusion

The findings of this study highlight the complex issue of CSMR and the various perspectives from which it can be viewed. While individual medical risks associated with CSMR may be perceived as low, the population-level risk may increase as the prevalence of CSMR rises. Perceptions of risk vary depending on the perspective from which they are viewed. It is crucial to balance individual autonomy with broader population-based perspectives. Both medical and psychological risks should be considered in the decision-making process concerning CSMR, and a holistic approach may be beneficial to ensure the long-term well-being of the woman, her child, and society. Stakeholders view counselling as essential in the decision-making process for CSMR, yet it is known that many women perceive a planned CS as a straightforward solution without the need for counselling. This discrepancy can create a conflict between women’s autonomy in making decisions about their bodies and healthcare professionals’ obligations and assessments. Continuity of midwife care models emerge as a potential alternative to CSMR for some women. These models can enhance control, trust, and reduce fear, potentially decreasing the number of primarily multiparas considering CSMR. Offering alternatives within the Swedish maternity care system, of which continuity of midwife care models and CSMR are integral parts, can contribute to a more individualised maternity care system. For some women, CSMR may be the optimal solution, while for others, different approaches may be viable. However, changes in Swedish maternity care are necessary to make the provision of such alternatives, and thereby a more individualised maternity care system, feasible. Stakeholders have a central responsibility for the organisation and direction of maternity care, along with the capacity to influence its development. By including their views, broader insights into the aspects of CSMR were obtained. Their perspectives highlight the need for clear guidelines, equal care, and strengthened trust in the maternity system.

## Data Availability

The datasets generated and/or analysed during the current study are not publicly available for reasons of confidentiality but are available from the corresponding author on reasonable request and with the permission of the Ethical Review Authority.

## Abbreviations

CS: Caesarean Section
CSMR: Caesarean Section on Maternal Request
NICU: Neonatal Intensive Care Unit
WHO: World Health Organisation
